# Veterinary-prescribed physical activity promotes walking in healthy dogs and people

**DOI:** 10.1186/s12917-020-02682-z

**Published:** 2020-12-01

**Authors:** Colleen Duncan, Angela Carswell, Tracy Nelson, Dan J. Graham, Felix M. Duerr

**Affiliations:** 1 Department of Microbiology, Immunology and Pathology, College of Veterinary Medicine and Biomedical Sciences, Colorado State University, 1601 Campus Delivery, Fort Collins, CO 80523 USA; 2 Department of Clinical Sciences, College of Veterinary Medicine and Biomedical Sciences, Colorado State University, 1601 Campus Delivery, Fort Collins, CO 80523 USA; 3 Department of Health and Exercise Science and Colorado School of Public Health, College of Health and Human Sciences, Colorado State University, 215D Moby Complex B, Fort Collins, CO 80523 USA; 4 Department of Psychology, College of Natural Sciences, Colorado State University, 1876 Campus Delivery, Fort Collins, CO 80523 USA

**Keywords:** Exercise prescription, Physical activity, Dog walking, Wearable device

## Abstract

**Background:**

Regular physical activity (PA) promotes health and can prevent and treat diseases among both humans and dogs. Unfortunately, most U.S. adults do not meet PA recommendations, and many dogs are also insufficiently active. Veterinary-prescribed PA programs have shown some success in increasing activity among overweight dogs, but the impacts of such programs have not yet been tested for efficacy among otherwise-healthy dogs and owners. In addition, although wearable devices that monitor PA and provide individuals with feedback (e.g., progress toward a daily step goal) can effectively increase human PA, it is unclear what impact similar wearable devices have on human and dog PA when the PA-monitoring devices are worn by dogs. The present study assessed the impact of an 8-week veterinary-prescribed PA program on activity and health among dogs and their owners, and randomized participants (*n* = 59) to two groups: one in which PA was measured but not visible to participants (*n* = 30), and one in which PA was measured and real time feedback was visible through a wearable device (*n* = 29).

**Results:**

Participants in both groups showed significant PA increases over the course of the 8-week program. Biomedical testing performed at the veterinary clinic facilitated early diagnosis of systemic illness in one human participant. The frequency of hypertension in human participants decreased significantly from baseline to the end of the program (week 8). Other health indices (e.g., BMI in humans, body weight and BCS in dogs) improved, albeit not to a statistically significant extent, over the course of the program. There were no significant differences on the outcomes of interest between the two experimental conditions.

**Conclusions:**

Veterinary-prescribed PA programs appear promising for increasing PA among insufficiently active but otherwise healthy dogs as well as their owners. Additional testing of veterinary-prescribed PA is warranted, particularly at other types of veterinary clinics (e.g., private practices). Incorporating wearable devices permitting owners to track canine PA did not appear necessary for obtaining these benefits; however, additional studies investigating alternative devices or different time periods may be warranted.

**Supplementary Information:**

The online version contains supplementary material available at 10.1186/s12917-020-02682-z.

## Background

Regular physical activity (PA) is an integral part of both health promotion and disease treatment in humans and animals alike [1–4]. The human health benefits of PA have been extensively reviewed with numerous studies highlighting the role of PA in preventing a wide range of diseases along with a reduction in the risk of premature death [1, 4, 5]. Similarly, in dogs PA and appropriate weight management is known to increase lifespan, improve quality of life, and reduce the incidence, severity, and clinical symptoms of osteoarthritis [3, 6–9].

Substantial health outcomes are realized even with relatively low levels of PA [4]. One of the simplest and most accessible forms of PA is walking, which has been made a national priority by the US Surgeon General [10]. Despite the known benefits and accessibility of walking, approximately half of the adult population in the US does not meet the minimum guidelines for PA recommended by the US Department of Health and Human Services of at least 150 min per week [11–15]. The role of dog ownership in the promotion of human PA is an increasingly important area of research. In general, dog ownership has been associated with increased PA and dog owners are more likely than those who do not own dogs to meet PA guidelines of at least 150 min per week [16–19]. Unfortunately however, only a subset (~ 60%) of dog owners routinely walk their dogs [17], and 59% of dogs are overweight [20] or have other health conditions that could be addressed by physical activity, suggesting that there is substantial room for increasing PA for both dogs and people. Strategies to increase dog walking would be beneficial for both dogs and people [21] and therefore, not surprisingly, this topic -promoting dog walking - is increasingly interesting for researchers and public health practitioners [22, 23].

Recent technology advances have resulted in wide availability of ‘pet wearables’; various canine activity trackers are available at reasonable cost to pet owners [24]. These devices offer features including the monitoring of location via GPS and vital parameters along with the quantification of activity. Activity data is measured by accelerometers, small electromechanical devices that can be used to calculate daily activity by measuring acceleration forces in one or more planes of movement. Accelerometry has been shown to be an effective way to obtain more detailed PA frequency, duration, and intensity data from people walking with their dogs [25]. Surprisingly, there is a paucity of research that utilizes this technology to provide objective outcome data on the benefits of animal-associated PA [17]. Furthermore, there is a lack of research investigating whether the real-time feedback provided by the applications motivates dog owners to become more active. In people, the use of accelerometers has been associated with significant increases in PA, > 25% over baseline, particularly when users can view their progress toward a daily ‘step goal’ [26]. In a 2020 review of the 13 available studies testing the impacts of dog-facilitated PA interventions, 5 utilized accelerometry, but none incorporated wearable canine devices that provided dog owners with visible PA data [19]. Thus, it is currently unknown if such wearable technology, when worn by dogs, results in a positive effect on PA promotion for dogs and/or their human walking partners.

Another well-established mechanism for promoting human PA has been through exercise prescription from primary care physicians [27]. Some work involving veterinary care has been performed suggesting that prescribing PA may be particularly useful for owners of dogs with medical conditions [28, 29]. However, it remains unclear what impact exercise prescriptions have in the field of disease prevention; specifically, whether exercise prescriptions are effective when given to dogs who are not currently meeting PA guidelines, but who are otherwise healthy.

The objective of this study was to determine if veterinary-prescribed PA could increase dog walking to meet PA guidelines among otherwise healthy dogs. A secondary objective was to determine if real-time feedback of activity data increased the levels of PA owners undertook with their dogs. We hypothesized that dog owners would be responsive to veterinarian prescribed PA and that the use of canine accelerometers capable of providing real-time activity monitoring and feedback would result in better compliance to a prescribed exercise plan for dogs, and improved health outcomes in both people and dogs.

## Results

**Fig. 1:**
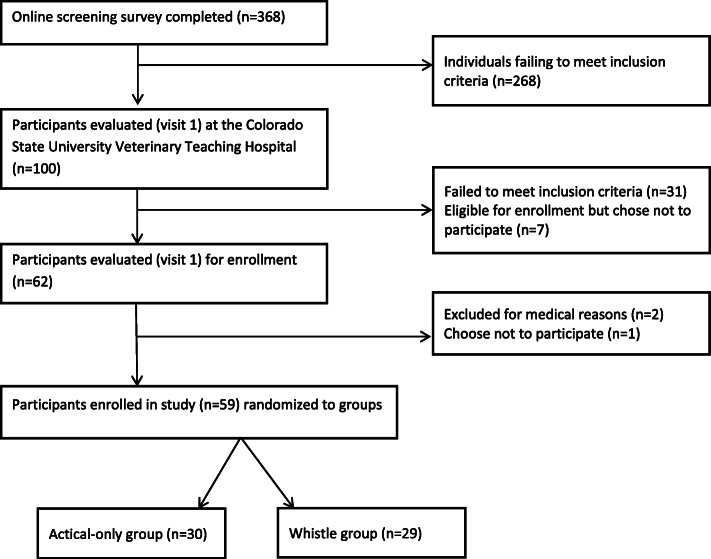
Schematic representation of the recruiting and enrollment process for study participants

The study was conducted between November 2017 and November 2018. An overview of screening and enrollment is presented in Fig. 1. A total of 59 owner-dog pairs initiated the 8-week walking program, with 30 participants in the Actical-only group and 29 in the Whistle group. Characteristics of the two groups are summarized in Table 1; there were no statistically significant differences in mean age, weight, body condition score (t-test, *p* > 0.05 for all) or sex (chi-square test, *p* = 0.36) between groups. All exit data (activity monitoring, exercise log, exit questionnaire) were obtained from 35 (22 Actical-only and 13 Whistle) participants; activity data were unavailable from 4 dogs due to technical issues with the collar. Eight participants failed to complete the exit questionnaire and activity log, 6 the exit questionnaire only, and 6 the activity log only.

**Table 1:** Characteristics of dogs in the two study groups

	Actical-only (***n*** = 30)	Whistle (***n*** = 29)
Age (days)	2587 ± 1303	2528 ± 1430
Weight (kg) (mean ± SD)	22.6 ± 9.7	25.9 ± 12.6
BCS (1–9 scale)	5.5 ± 0.72	5.8 ± 0.75
Sex	9 male, 21 female	12 male, 17 female
Breeds	Mixed breed (14), golden retriever (3), border collie (2), boxer (2), Labrador (2), Australian shepherd (1), beagle (1), Carin terrier (1), schipperke (1), shih tzu (1), standard poodle (1), toy poodle (1)	Mixed breed (18), Labrador (2), Australian shepherd (1), Catahoula Leopard Dog (1), cocker spaniel (1), German shepherd (1), golden retriever (1), miniature dachshund (1), Siberian husky (1) Staffordshire terrier (1), Yorkshire terrier (1)

### Activity data

**Fig. 2:**
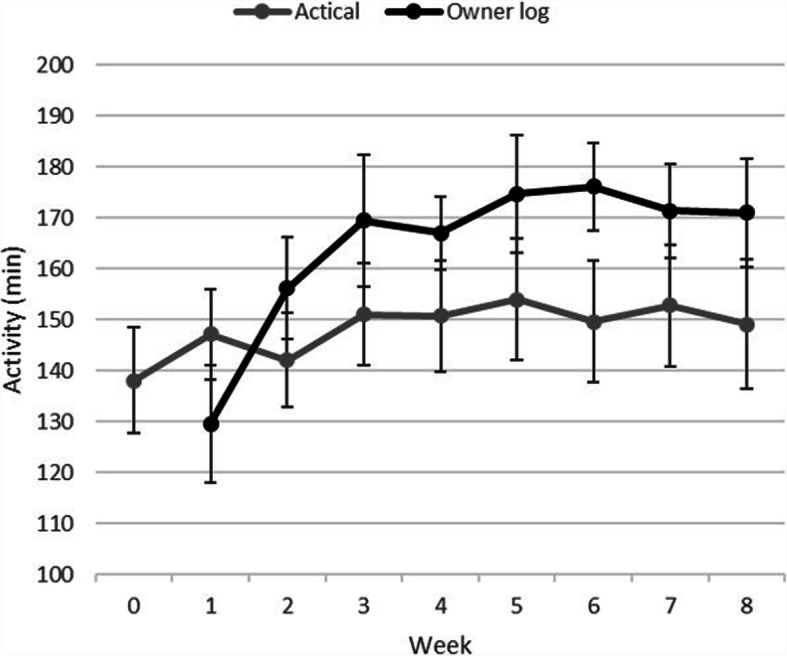
Activity data by week of the veterinarian prescribed walking program as reported by the owner in the activity log (‘owner log’ in minutes per week ± standard error) or measured by the collar mounted Actical accelerometer (‘Actical’ in average minutes per week classified as active (walking, trotting and vigorous activity combined) ± standard error)

The weekly collar-measured activity (Actical) and owner-reported PA is presented in Fig. 2. Overall participants exceeded the minimum prescribed PA for all weeks but followed the general recommended three-week build-up. There was no statistically significant difference in the owner-reported PA between the Whistle and Actical-only groups (t-test, *p* > 0.05 for all weeks). Similarly, Actical data showed a general increase in collar-measured PA with the mean activity count for the 8 weeks of prescribed exercise (146.96) that was statistically higher than baseline activity counts (138.12, paired t-test, *n* = 55, *p* = 0.019) but this also did not differ statistically between the two collar groups (t-test, *p* > 0.05 for all weeks).

### Human biometric data

Most participants agreed to have at least some of their own health measures taken and recorded at study entry (*n* = 52; 41 women and 11 men) and/or exit (*n* = 35; 28 women and 7 men). Seven people (5 women and 2 men) were classified as hypertensive at entry but only 4 (3 women and 1 man) were hypertensive at the time of exit screening, a difference that was statistically significant (Fisher’s Exact test *p* = 0.002). Dog owners participating in both entry and exit biometrics had a mean BMI that decreased over the study for both women (entry 28.3 ± 7.1, exit 27.2 ± 4.9) and men (entry 26.8 ± 4.7, exit 25.2 ± 4.4). This difference was not statistically significant (paired t-test *p* > 0.05 for both genders and combined) however one woman originally categorized as overweight (BMI > 25) was classified as normal weight upon recheck. One woman with a markedly elevated blood glucose (385 mg/dL) was consequently diagnosed as diabetic by her personal physician and at the 8-week exit screening her blood glucose had normalized. No significant differences were noted in the waist circumference, total cholesterol levels, LDL-C, HDL-C, or triglycerides between entry and exit (paired t-tests, *P* > 0.05 for all).

### Canine biometric data

The mean weight of all dogs (*n* = 59) decreased between visit 1 (24.5 kg ± SD 10.7) and visit 3 (23.6 ± 11.0) as did the body condition score BCS (BCS, from 5.5 to 5.4). When considering only those dogs who were overweight (having a BCS greater than or equal to 6) at baseline, canine mean weight also decreased from visit 1 (24.1 kg ± 11.1) to visit 3 (23.6 ± 10.6) with a concurrent decrease in BCS from 6.3 to 6.2. None of these results were statistically different at the *p* < .05 level (paired t-tests, *p* > 0.05 for all).

#### Owner questionnaires

Of the respondents in the Whistle group, 67% reported that the feedback from the Whistle activity monitor increased the duration and/or frequency of the time spent exercising with their dog. All of the remaining respondents (33%) reported that the feedback from the device did not change their behavior but that they increased their PA anyway. Within the Whistle group, 79% of the respondents reported having experience with a human activity tracker. The ability to visualize data was the most commonly reported positive feature of the device. The most commonly reported downsides were technical issues and battery duration, with 67% of respondents reporting some degree of technical difficulty and 50% of respondents reporting that they forgot to charge the device at least once. Overall, 83% of the respondents found the device and application to be worthwhile and 78% of respondents agreed that their experience with the device made them more likely to purchase one for their own use. Of the respondents in the Actical-only group, 76% reported that knowing the research team was receiving feedback on their dog’s activity caused them to increase the exercise they did with their dog. Fifty-two percent of the Actical-only group respondents reported having experience with a human activity tracker that provides real-time feedback and 69% of the respondents thought that a similar activity tracker would work for their dog.

## Discussion

Results of this study suggest that a veterinary-prescribed exercise plan for dogs can increase PA for dogs and owners who are not meeting recommended PA levels. This finding is significant as this study is the first to target canine physical inactivity. Previous studies had only investigated the impact of veterinary-prescribed physical activity targeting animal [28] or human [29] obesity. Byers et al., in their study of overweight dogs, found an improvement in some human and animal health metrics when PA was prescribed by a veterinarian; however, no significant increase in objectively measured PA of the dog owners was recorded [28]. Kushner et al. showed that PA for combined weight loss (i.e. if dogs and owners are both overweight) can be effective [29]. As the health benefits of PA can be preventative as well as therapeutic, the goal of our work was to determine the effectiveness of veterinary-prescribed PA for pets not currently meeting PA guidelines, i.e. our approach represents a prevention- instead of treatment-focused approach. In the United States, approximately 40% of dogs are not regularly walked [17]. Strategies to increase dog walking are important for two reasons: improving human health and improving dog health. With an estimated dog population of ~ 90 million in the U.S. alone [30], the potential for veterinarians to have a significant public health impact is vast.

The number of dog owners willing to have their own biometric data collected by a veterinary technician at a veterinary hospital was surprising. The overwhelming majority of participants elected to have at least some of the offered testing conducted and for one participant this screening led to a diagnosis of a previously unknown, and serious, medical condition. Opportunities for individuals to access health screening services at convenient locations has been long advocated. Effective campaigns have been provided through non-medical industries such as salons and barbershops [31] or through non-physician medical professionals such as pharmacists [32]. While veterinarians are involved in several public health efforts such as zoonotic disease control and food safety, the potential for clinics to serve as community health screening centers has not been explored. However, this approach may provide a solution to address the disparities in access to quality health care services in rural as compared to urban areas [33].

Given the short study period, significant changes in biometrics were not anticipated. Yet, some important trends were documented: measures of body condition in dogs (i.e., weight and BCS) and their owners (i.e., BMI) and the frequency of owner hypertension decreased throughout the walking program. This is a notable finding, since weight loss was not specifically targeted, and no other weight-associated interventions were included. In this study more than 90% of participants reported that they expected to continue walking after completing the 8-week program and that the program led them to change other health-associated behaviors for them and their dog (data not shown). This is encouraging since a longer study period likely would have identified greater improvements in health metrics. Our study shows feasibility that such measurements can be conducted within the veterinary community.

Several approaches to encourage dog walking amongst dog owners have been investigated but met with limited success [15, 34, 35]. One objective of our study was to investigate whether real-time feedback from canine activity trackers may provide a motivation for dog owners to increase their dog-associated PA; however, we were unable to show an improved compliance with the exercise plan with the use of pet wearables. While feedback from activity monitors (e.g. Fitbit) has been shown to increase PA in people [26], it is possible that this effect does not translate when the device is worn by pets. Alternatively, our study may have been unable to detect a small difference, if one was present, due to low power or the potential ceiling effect given the excellent compliance in both groups. In addition, the participants in both groups likely already possessed a high degree of motivation to walk, given that they enrolled in this walking study. Such motivation may decline over time such that differences between groups may have been identified in a study of longer duration. Of the respondents in the Actical-only group, 76% reported that knowing the research team was receiving feedback on their dog’s activity caused them to increase exercising their dog. As such, their pet wearing an activity tracker (regardless of whether feedback was provided) may have already increased the amount of time spent walking, and being able to see the activity data themselves on a separate device may not have added much to the increased motivation from knowing the activity data were being recorded and later viewed by the study team. As the exercise prescription in this study was total minutes per week, it is also possible that the feedback from the Whistle device, which included all of the dog’s daily activity - including PA completed without the owner - was not as relevant to the owner as feedback from Fitbits and other human wearables previously demonstrated to influence behavior. While feedback on the device was generally positive, the frequency or duration of owner engagement with the feedback application was not measured. We were therefore unable to determine how often the data were accessed and analysis may have included owners that did not (fully) utilize the feedback. Additionally, several owners experienced technical difficulties with the set-up. Given these factors and the increasing number and decreasing costs of wearable activity monitors for both humans and pets, the authors believe that our study results should not deter future research in this area.

Consistent with the Rhodes et al., 2020 review of dog-facilitated PA interventions, the present study supports the feasibility and potential effectiveness of veterinary-prescribed PA programs and extends the literature by demonstrating this effectiveness even among a sample of insufficiently active, but otherwise-healthy dogs. Despite the promising results observed in this study, additional work is needed prior to wide-scale adoption of programs like the one tested here. Although participation in this project was not restricted to clients, this project was conducted in an academic veterinary teaching hospital that is different than many private practices; pilot testing of a similar program in private practice would be helpful. There was also no cost to the dog owners enrolled in this study, so an understanding of an individual’s willingness to pay for the program, or integration of the program within existing wellness plans, must be considered. This study failed to control for human demographic factors that may influence dog-walking behavior and additional research should explore the receptiveness of different dog-owning cohorts to veterinarian-prescribed PA. Similarly, this study relied on owner reported walking data which may introduce bias; future studies should include objective activity monitoring in humans as well as dogs. Finally, the 8-week walking program used in this study was informed by PA recommendations for people [10] and dogs [36]; however there are likely other PA promotion strategies that could be used by veterinarians. Future studies should investigate the impact of different PA programs on both short- and long-term health impacts.

## Conclusion

The present study outlines several novel ideas addressing the call for a One Health approach [37] to attend to the shared human/pet concern of overweight and obesity. Among otherwise healthy, but insufficiently active dogs and their human owners, an eight-week, dog walking program resulted in significant increases in physical activity for both dogs and owners. Measures of both human and dog health demonstrated favorable trends, and dog/human pairs in which the owner could view PA data on a canine wearable device obtained the same outcomes as those pairs without devices. Veterinary-prescribed PA programs appear promising and should be tested in additional settings.

## Methods

### Inclusion criteria

Dog owners were recruited in Fort Collins, Colorado through advertisements in the local newspaper, pet stores, radio stations, and social media. Participation in the study was incentivized by providing study-associated examinations and bloodwork at no cost to study participants. Dog-owner pairs that met the following inclusion criteria were eligible for enrollment; dogs had to: be systemically healthy (i.e. enrolling veterinarian deemed that 30 min of walking per day would be feasible for patient); have not used an activity monitor within the last 3 months; have not been walking 30 or more minutes/day for ≥5 days/week; and be leash-trained or walk off-leash only. Dog owners needed to own a smartphone with Bluetooth capability, have access to home WiFi, be physically able to walk 30 min/day, and agree to be the primary dog exerciser for the duration of the study (others were allowed to walk participating dogs but this would be in addition to the 30 min/day dogs walked with the primary walker). Interested owners had to complete an online survey to verify inclusion criteria; those that met the inclusion criteria were scheduled for a pre-enrollment visit at Colorado State University’s Veterinary Teaching Hospital.

### Visit 1 (pre-enrollment visit)

The purpose of this visit was to review the owner inclusion criteria, obtain consent from the owner, collect blood from the dog for a complete blood count and serum biochemistry that could be used as a component of the health assessment and fit the dog with the Actical activity device.^1^ Actical placement was performed to ensure the dog would tolerate the collar and to obtain baseline activity monitoring. Each dog was fitted with a collar^2^ so that two fingers could comfortably fit between the neck and the collar, but the collar could not be pulled over the head. The metal ring used for leash attachment was removed from each collar to avoid interference with activity data accuracy. The Actical device was chosen because it is an established small, lightweight, reliable activity tracking device that has been validated as an objective method for measuring canine PA [38, 39]. The Actical was firmly attached to the collar with two zip ties [38–40], positioned ventral to the mandible of each dog. The Actical epoch length was set to 60 s. The voluntary option of providing human health metrics at the subsequent appointment was discussed. Finally, the dog’s body weight was obtained.

### Visit 2 (enrollment visit)

Approximately one week later, owners returned for their 2nd visit and formal enrollment. At this visit participants were randomized into one of two groups by pulling one of two color chips from a bag that contained an equal number (*n* = 50) of each color. The ‘Whistle’ group was provided with a commercially available canine tracker^3^ while the ‘Actical-only’ group did not receive this additional tracker. The Whistle monitor provides real-time feedback regarding the intensity and duration of activity through a smartphone application (Whistle™). This monitor was attached to the collar, set-up with a WiFi link (to be used by the owners at home for set-up) and demonstrated to the owners. Both groups maintained the previously placed Actical monitor; as such, dogs in the Whistle group were equipped with two monitors and dogs in the Actical-only group with only one. Owners randomized to the Whistle group were provided with an additional consent form including information about the Whistle device. Dogs underwent a full physical examination (including assignment of a body condition score, BCS, which ranges from 1 (too thin) to 9 (too heavy) [41]) and any abnormal findings were discussed with the owners. Dogs with examination findings necessitating treatment or inability to participate in the walking program were excluded from the study. All participants then received one handout describing the importance of exercise in people and one infographic showing the combined human and animal benefits of dog walking. Participants in both groups then received a veterinarian-prescribed an eight-week walking program that included a 3-week build up to a total of 150 min of physical activity per week (Table 2). In addition, owners were provided a daily activity log and were asked to fill out an entry questionnaire.

**Table 2:** Weekly walking prescription for participants and their dogs

Week of Program	Total Minutes per Day (minimum)	Total Days per Week (minimum)
1	15	5
2	20	5
3	25	5
4–8	30	5

At this second appointment, owners were asked if they wanted to participate in the human health metrics portion, allowing measurement of any of the following: body weight, height, calculated body mass index (BMI), waist circumference (WC), and blood pressure. A commercially available, portable blood analyzer^4^ was utilized to measure glucose, total cholesterol, low-density lipoprotein cholesterol (LDL- C), high-density lipoprotein cholesterol (HDL-C) and triglyceride levels from blood obtained via fingerprick.^5^ All measurements were taken by a registered veterinary technician after completing the required institutional biosafety and human subjects training and written consent was obtained from all participants. The results were provided to the owners together with a handout including general information about normal and abnormal values and a recommendation to visit a physician with any questions.

### Visit 3 (exit visit)

Approximately eight weeks after visit 2, owners were asked to return for the exit visit. During this visit, owners were asked to fill out an exit questionnaire, the activity log was collected, and all devices (Acticals and Whistles) were returned. On the exit questionnaire there were a series of questions specific to the activity monitors which differed between the Whistle and Actical-only groups (Additional file 1). A full physical examination of the canine participant was performed, and owners were again offered to obtain their biometric data as during visit 2. Data from the Acticals were downloaded using the included hard- and software then raw data were imported into an Excel spreadsheet.

#### Data analysis

*Actical data* were processed within Excel to quantify the degree of activity. Cut points to differentiate the activity intensity for each minute were defined based on previous research [42]: 0–204 activity counts/minute = sedentary/recumbent; 205–1751 activity counts/minute = walking; 1752–6067 activity counts/minute = trotting; > 6068 activity counts/minute = vigorous. The number of minutes spent in each category was calculated for each day. Only days with complete data (i.e. 24 h) were included. Additionally, the number of minutes assigned a 0 was counted for each day and if > 1300 min were identified, data for this day were excluded from analysis (since this indicates that the collar was not placed on the dog). The average amount of minutes per week spent in an active stage (i.e. walking, trotting, and vigorous activity levels combined) was calculated. Only weeks with > 3 days were included in the data analysis. For baseline data, only the first week was calculated, even if owners spent multiple weeks before returning for visit 2. Calculation of week 1 data was initiated for the first full day after visit 2, to allow completion of a full day within the walking program. Similarly, the data from visit 3 were excluded from analysis. If owners completed an activity log, the log start and end date were used to determine start and end date of the walking program.

Descriptive and comparative statistics were conducted using commercially available software.^6^ Physical activity data were compared between groups using t-tests. Entry and exit human and dog biometrics were compared using paired t-tests and Fisher’s Exact test for frequencies. Participants were classified as hypertensive if their systolic blood pressure was greater than or equal to 140 mmHg or their diastolic blood pressure was greater than or equal to 90 mmHg.

## Supplementary Information

**Additional file 1.** Activity Monitor Review Questions from Exit Questionnaire.

## Data Availability

The datasets used and/or analyzed during the current study are available from the corresponding author on reasonable request.

## Abbreviations

PA: Physical activity
BCS: Body condition score
BMI: Body mass index
WC: Waist circumference
LDL-C: Low-density lipoprotein cholesterol
HDL-C: High-density lipoprotein cholesterol

## Footnotes

1 Respironics Mini Mitter Division, Bend, OR

2 Lupine Basics, Conway, NH

3 Whistle™ W01A, Whistle Labs, Inc., San Francisco, CA

4 Alere Cholestech LDX Analyzer, Alere, Inc., San Diego, CA

5 Acti-Lance 1.8 mm 23G Safety Lancets, HTL-STREFA, Inc., Marietta, GA

6 https://www.ibm.com/analytics/spss-statistics-software
