# Supplementary material for: Veterinary-prescribed physical activity promotes walking in healthy dogs and people
Source: BMC Vet Res. 2020 Dec 1;16:468. doi: 10.1186/s12917-020-02682-z (PMC7709226; doi:10.1186/s12917-020-02682-z)
Supplement: Supplementary file 1 — Additional file 1. Activity Monitor Review Questions from Exit Questionnaire. [file 12917_2020_2682_MOESM1_ESM.docx]

**Additional file 1: Activity Monitor Review Questions**

**Activity monitor review questions: Actical-only group**

1. Did knowing the research team was receiving feedback of your dog’s activity cause you to change the frequency (number of times per day or per week) or duration (amount of time) you and your dog exercised together?

- Yes (please check the best reason for your ‘Yes’ answer)
  - we *increased* the amount of our exercise together because I knew the research team was receiving feedback
  - we *decreased* the amount of our exercise together because I knew the research team was receiving feedback
- No (please check the best reason for your ‘No’ answer)
  - the amount of our exercise together *stayed the same* as before the study
  - we changed the amount of our exercise together, but *not due to knowing the research team was receiving feedback*

2. Do you personally have experience with a human activity tracker (such as FitBit or Jawbone)?

- Yes
- No

Do you think a similar activity tracker would work for your dog?

- Yes
- No

**Activity Monitor Review Questions: Whistle group:**

- 1. Did the feedback from the Whistle activity monitor cause you to change the frequency (number of times per day or per week) or duration (amount of time) you and your dog exercised together?
  - Yes (please check the best reason for your ‘Yes’ answer)
    - we *increased* the amount of our exercise together as a result of the feedback from Whistle
    - we *decreased* the amount of our exercise together as a result of the feedback from Whistle
  - No (please check the best reason for your ‘No’ answer)
    - the amount of our exercise together *stayed the same* as before the study
    - we changed the amount of our exercise together, but *not* *due to the feedback* from Whistle
  1. What was your favorite thing about using the Whistle activity tracker and app? (open text)
  2. What was your least favorite thing about the Whistle tracker and app? (open text)
  3. Did you experience any technical difficulties using the Whistle activity tracker and app?
- No
- Yes

If yes, please describe: (open text)

- 1. How often did you forget to charge the Whistle activity tracker *(i.e.* the device was without charge for more than 12 hours)?
- Never
- Once during the study period
- 2-3 times during the study period
- More than three times during the study period
  1. Did you find use of the Whistle activity tracker and app worthwhile?
- Yes
- No
  1. Did your experience with the Whistle activity tracker and app make you more likely to purchase one for personal use with your dog?
- Yes
- No
  1. Is there anything you would change about the Whistle activity tracker and app? (open text)

9. Do you personally have experience with a human activity tracker (such as FitBit or Jawbone)?

- Yes
- No
